# Methanol Poisoning: An Autopsy-Based Study at the Tertiary Care Center of Uttarakhand, India

**DOI:** 10.7759/cureus.25434

**Published:** 2022-05-28

**Authors:** Vikas Vaibhav, Pawan K Shukla, Raviprakash Meshram, Ashish R Bhute, Abhishek Varun, Prashant Durgapal

**Affiliations:** 1 Forensic Medicine and Toxicology, All India Institute of Medical Sciences, Rishikesh, IND; 2 Forensic Medicine, All India Institute of Medical Sciences, Raipur, IND; 3 Forensic Medicine, All India Institute of Medical Sciences, Rishikesh, IND; 4 Forensic Medicine, All India Institute of Medical Sciences, New Delhi, IND; 5 Pathology, All India Institute of Medical Sciences, Rishikesh, IND

**Keywords:** hooch tragedy, septicemia, toxic neuropathy, metabolic acidosis, optic neuropathy, methanol poisoning

## Abstract

Aims and objective

This study describes postmortem and histopathological findings to understand the internal progression of methanol poisoning. The study also aims to examine clinical, biochemical, and histological changes seen with methanol poisoning.

Materials and methods

The study describes the methanol poisoning tragedy that occurred in February 2019 in the Haridwar district of Uttarakhand. Ninety-one patients were admitted to the hospital, four were brought dead, four died within a few hours of admission (designated as early deaths), and four died between 10 and 45 days of hospitalization (designated as late deaths). A medicolegal autopsy was performed on all 12 deaths. Gross external and internal findings were noted, and routine viscera and blood were preserved and sent to Uttarakhand's Forensic Science Laboratory (FSL) to estimate methyl alcohol. A section of the optic nerve was taken from the optic chiasma for histopathological examination. Data were collected retrospectively from records. All data were tabulated and analyzed using Microsoft Excel version 2019. The study was approved by the Institutional Ethics Committee of All India Institute of Medical Sciences (AIIMS), Rishikesh (249201), Uttarakhand, India.

Results

Methanol poisoning is a health-associated disaster in many regions of India. Autopsy, including histopathological examination, could elicit the adverse effects of methanol on different organs. Their mean age was 37.7 years (range 21-70), and 67% (n = 8) of all victims were in the age range of 30-50 years. The average methyl alcohol level reported among hospital deaths and brought dead was 116.08 mg/dl and 224.6 mg/dl, respectively. A blurred vision had been their most common complaint, identified in 75% (n = 6), followed by vomiting and abdominal pain, while 50% (n = 4) had features of respiratory insufficiency. Mean pH and bicarbonate levels among hospital deaths were 6.61 mmol/l and 6.18 mmol/l, respectively. An autopsy revealed signs of hypoxia in all cases. Internal organs were congested. Severe metabolic acidosis leading to the respiratory failure was the cause of death in early deaths. Cerebral and pulmonary edema consequent upon septicemic shock was the cause of late deaths. A case with the most extended survival duration showed cerebral edema with intracerebral hemorrhage. Cirrhotic liver, along with features of renal failure, was an additional internal finding in late deaths. Optic nerve histopathology showed no demyelination or axonal necrosis; however, mild edematous changes were evident.

Conclusions

Methanol poisoning is one of the manmade disasters in the developing world. There are various adverse effects of different organs and organ systems inside the body. Timely intervention and diagnosis can save several lives. The organ-directed meticulous autopsy can help autopsy surgeons in establishing the diagnosis and thus help the judiciary in delivering justice to the sufferers.

## Introduction

Methanol poisoning, sometimes described as a hooch tragedy, is widespread in underdeveloped and developing nations [1,2]. The word hooch is said to be derived from the Hoochinoo Indians in the 18th century. They are a small tribe in Southern Alaska who used to make illicit alcohol for their consumption, and this practice got so popular that homemade alcohol was dubbed “hooch in distinct cultures” [3].

In India, multiple incidents of methanol poisoning have been reported in the past, generally involving people from lower socioeconomic groups [4,5]. Methyl alcohol is illegally prepared and sold as a replacement for expensive, unaffordable ethanol. India has a booming illegal alcohol industry, and over the last three decades, more than 2,000 people have died [6].

The central organ of methanol metabolism is the liver. Methyl alcohol is first oxidized to formaldehyde with the help of alcohol dehydrogenase, which is further metabolized to formic acid via aldehyde dehydrogenase [7]. These metabolites are responsible for the most adverse effects of intoxication. There is usually a lag between the ingestion of methanol and the appearance of symptoms due to a delay in forming these metabolites [7]. The diagnosis of intoxication may go unnoticed due to this latency.

Ophthalmological complications, blindness, optic neuropathy, brain edema, acute renal failure, and severe metabolic derangement are frequently encountered toxic complications [4,5,7,8]. Many studies and case reports are available to rationalize the management protocol for early diagnosis and treatment [9-12]. However, studies comparing the clinical and biochemical profile with postmortem findings of methanol poisoning cases are limited. This study describes the postmortem and histopathological findings to understand the internal progression of methanol poisoning. The study also aims to examine clinical, biochemical, and histological changes seen with methanol poisoning.

## Materials and methods

Sample size

This study describes the methanol poisoning tragedy that occurred in February 2019 in the Haridwar district of Uttarakhand. It involved more than 200 people, of which 31 people died and 169 people survived following treatment.

Procedure

Of 91 patients admitted to the hospital, four were brought dead, four died within a few hours of admission (designated as early deaths), and four died between 10 and 45 days of hospitalization (designated as late deaths). The deaths were divided into two categories: hospital deaths (i.e., brought in alive but died during treatment; n = 8) and brought death (n = 4). A medicolegal autopsy was performed on all 12 deaths. Gross external and internal findings were noted, and routine viscera and blood were preserved and sent to Uttarakhand's Forensic Science Laboratory (FSL) to estimate methyl alcohol. A section of the optic nerve was taken from the optic chiasma for histopathological examination.

Data collection

Data were collected retrospectively; hospital records were reviewed to obtain relevant information, including incidence history, clinical examination, biochemical profile, treatment, and outcome. Relatives of the deceased who were brought dead were also interviewed to get relevant details.

Analysis and ethical approval

All data were tabulated using Microsoft Excel version 2019. This study was approved by the Institutional Ethics Committee of All India Institute of Medical Sciences (AIIMS), Rishikesh (249201), Uttarakhand, India, Letter no# AIIMS/IEC/20/342.

## Results

All of the deceased were men and were of low socioeconomic status. Their mean age was 37.7 years (range 21-70), and 67% (n = 8) of all victims were in the age range of 30-50 years. All of the deceased were brought to our center within 24 hours of methanol ingestion. The average methyl alcohol level reported among hospital deaths and brought dead was 116.08 mg/dl and 224.6 mg/dl, respectively (Tables 1, 2).

**Table 1:** Demographic description of methanol poisoning cases

S. No.	Demographic parameters	Values
1	Total reported methanol poisoning cases	91
2	Total deaths	12
	Brought dead	04
	Hospital death	08
3	Mean age of all deaths in a year (minimum-maximum)	37.7 (21-70)
4	The most affected age group in years	35-50, 67% (n = 8)
5	Gender (Male)	100% (n = 12)
6	Mean postmortem interval	13.5 hours

**Table 2:** Clinical profiles of methanol poisoning cases FSL: Forensic Science Laboratory.

Clinical parameters		Hospital deaths (n = 8)	Brought dead (n = 4)
1.	The average methanol level as reported by FSL in mg/dl	116.08	224.6
2.	Underwent hemodialysis	100%	N/A
3.	Clinical symptoms		
	Neurological	100%	N/A
	Ophthalmological	75% (n = 6)	N/A
	Gastrointestinal	37.5% (n = 3)	N/A
	Respiratory	50% (n = 4)	N/A
4.	Mean arterial pH	6.61	N/A
5.	Mean bicarbonate level in mmol/l	6.18	N/A

Among hospital deaths, all patients were brought in a coma. The blurred vision had been their most common complaint, identified in 75% (n = 6), followed by vomiting and abdominal pain, while 50% (n = 4) had features of respiratory insufficiency. Mean pH and bicarbonate levels among hospital deaths were 6.61 mmol/l and 6.18 mmol/l, respectively. Biochemical investigations revealed features of acute renal failure among those who died in the hospital (Tables 1, 2).

**Figure 1:**
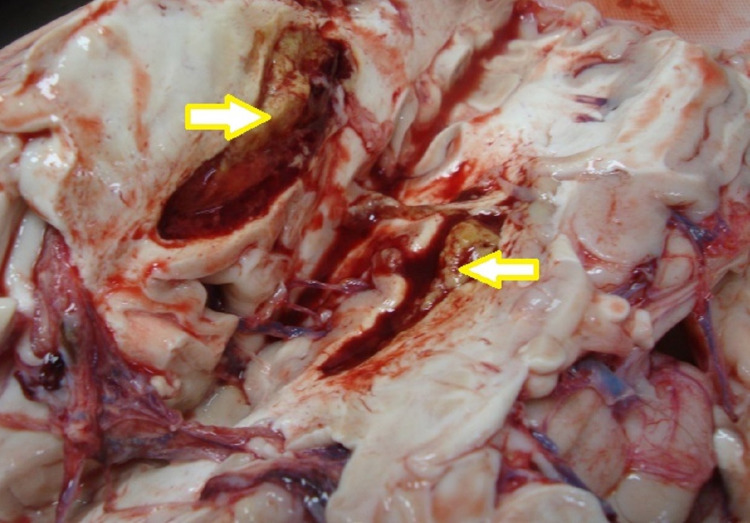
Intracerebral hemorrhage (shown by yellow arrows) involving bilateral basal ganglia and the intra-thalamic region surrounded by necrotic tissues seen on the sagittal section of the cerebrum

The mean postmortem interval was 13.5 hours, and an autopsy revealed signs of hypoxia in all cases. Internal organs were congested, and the average weight of the brain and lungs in overall deaths was increased (Table 3). Severe metabolic acidosis leading to the respiratory failure was the cause of death in early deaths. Cerebral and pulmonary edema consequent upon septicemic shock was the cause of late deaths. A case with the most extended survival duration showed cerebral edema with intracerebral hemorrhage involving bilateral basal ganglia and the intra-thalamic region, surrounded by necrotic tissues (Figure 1).

**Table 3:** Mean weight of brain and both lungs

Mean weight of brain and lungs among	Brain (in gm)	Lungs (in gm)
Total deaths (n = 12)	1463.7	1344.3
Early deaths (n = 8)	1395	946.2
Late deaths (n = 4)	1532.5	1742.5

**Figure 2:**
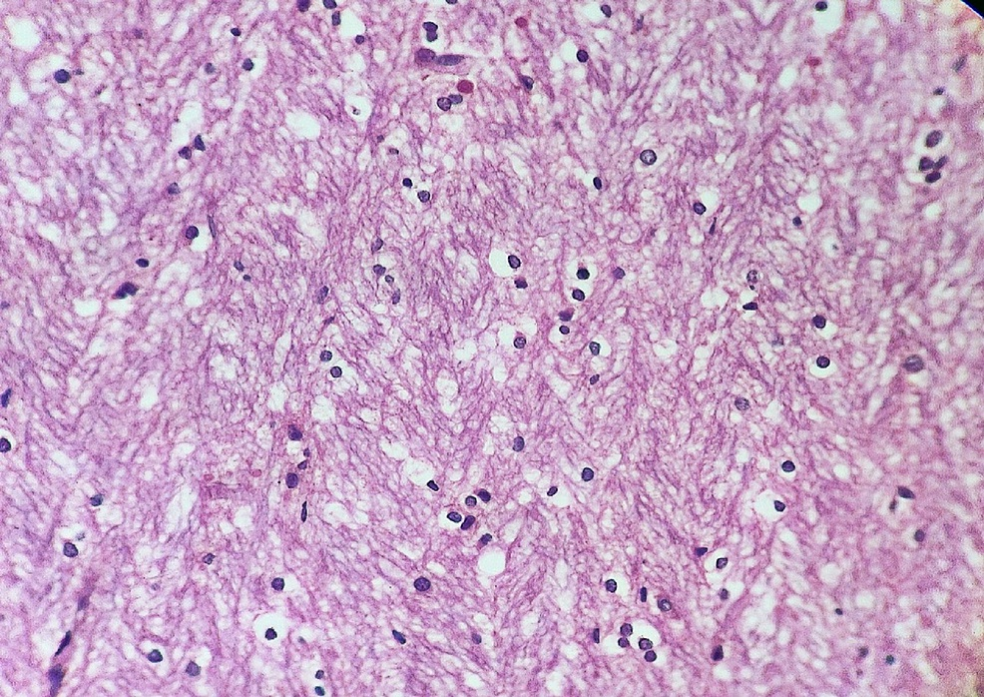
A section examined from the optic nerve showed mild edema. No demyelination or axonal necrosis was identified in the sections examined (Hematoxylin and eosin stain, magnification: 400x).

Cirrhotic liver, along with features of renal failure, was an additional internal finding in late deaths. Optic nerve histopathology showed no demyelination or axonal necrosis; however, mild edematous changes were evident (Figure 2).

## Discussion

This research adds to our understanding of the acute effects of methanol poisoning by evaluating and comparing the clinical and autopsy profiles of deceased persons. The primary cause of mortality in early deaths was metabolic acidosis leading to respiratory failure. Sepsis leading to multi-organ failure was the primary cause of late deaths. Formic acid, the oxidation by-product of methyl alcohol, is responsible for metabolic acidosis. As metabolic acidosis increases, mortality also increases [4,5].

The mean age of victims in this study was 37.7 years. Males between 30 and 50 years, belonging to low socioeconomic groups, were the most affected. These findings were similar to other studies showing middle-aged men are the most likely to become victims [1,2,7]. The average methanol level among hospital and brought deaths were 116.08 mg/dl and 224.6 mg/dl, respectively. However, according to several studies, the fatal methanol level may vary. The minimum and maximum fatal levels reported in a study were 73 and 326 mg/dl, respectively [9], while further research reported a range of 60-200 mg/dl as the fatal blood level for methanol [2]. The same methanol level may have a variable outcome in different people [10]. This could be attributed to a number of factors such as differences in demographic profiles, comorbid conditions, tolerance levels, and metabolic rates.

The most common symptoms reported by victims of methanol poisoning are gastrointestinal [4,11], visual disturbances [5], and neurological phenomena [12]. In the present study, all of the hospital deaths presented with coma. Based on the history provided by accompanying people, 75% (n = 6) had visual symptoms, followed by respiratory (50%; n = 4) and gastrointestinal (37.5%; n = 3). As per available evidence, most patients who died in hospitals presented with coma or shortness of breath [4,5,11]. Frequent neurological features encountered in clinical settings include headache, vertigo, altered sensorium, and coma [12]. Studies describe the brain and kidney as the most frequently affected organs contributing to death. Cerebral edema, congestion, intracerebral hemorrhages, and degenerative necrosis are the known central nervous system findings. Other common findings are pulmonary edema, superficial hemorrhages of the lungs, and fatty changes in the liver. Renal damage may also be evident as patchy tubular degeneration with necrosis [1,2,13].

The findings of our study are consistent with the characteristics described above. Cerebral and pulmonary edema were observed in all cases but were more apparent in late deaths, and the increased weight of the brain and lungs confirmed the same (Table 3). Intracerebral hemorrhage involving basal ganglia and thalamus was present in the longest surviving individual, while fatty changes were evident in older victims. Histopathology of the optic nerve from optic chiasma showed mild edematous changes without demyelination or axonal necrosis. However, significant demyelination with intra-axonal swelling and organelle destruction is reported in a few studies [14,15].

A significant limitation of our study was the relatively small sample size. We could have measured the levels of ethanol, methanol, and its metabolites simultaneously, which could have explained the diverse clinical characteristics of victims more efficiently.

## Conclusions

Methanol poisoning is a health-associated disaster in many regions of India. Autopsy, including histopathological examination, could elicit the adverse effects of methanol on different organs and also assist the judiciary in providing justice to the sufferer. Ophthalmic, cerebral, pulmonary, and renal systems are commonly affected by methanol poisoning. Features of cerebral edema, pulmonary edema, and renal damage are to be looked for during autopsy and in the emergency department. Intracerebral hemorrhages could be an additional finding. The public must be educated and made aware of the consequences of adulterated methanol consumption, and the government should take strict steps to regulate the illegal liquor sector.
